# Nanoscale Insights into the Mechanical and Tribological Properties of a Nanocomposite Coating

**DOI:** 10.3390/nano15161280

**Published:** 2025-08-19

**Authors:** Chun-Wei Yao, Ian Lian

**Affiliations:** 1 Department of Mechanical Engineering, Lamar University, Beaumont, TX 77710, USA; 2 Department of Biology, Lamar University, Beaumont, TX 77710, USA; ilian@lamar.edu

**Keywords:** nanocomposite, nanoscale mechanical analysis, nanoscratch, metrology

## Abstract

This study investigates the mechanical and tribological behavior of a polydimethylsiloxane (PDMS)–silica nanocomposite coating over the temperature range extending from 24 °C to 120 °C. Nanoindentation tests revealed depth- and temperature-dependent variations in hardness and complex modulus. A time-dependent deformation model accurately captured the viscoelastic and viscoplastic behavior observed during sustained loading, providing predictive insight into the coating’s thermomechanical performance. Tribological evaluation through friction and nanoscratch testing demonstrated a temperature-induced increase in the coefficient of friction. The integration of mechanical and surface metrology and characterization techniques offers a comprehensive understanding of the coating’s behavior under thermal and mechanical stress. These findings support the design of robust nanocomposite coatings with superior functional performance for practical applications requiring enhanced mechanical stability, wear resistance, and thermal tolerance in challenging service environments.

## 1. Introduction

Nanocomposite coatings, consisting of nanoscale fillers dispersed within a polymer matrix, have emerged as a promising class of materials for advanced surface engineering due to their superior mechanical, thermal, and chemical properties [1]. A key advantage of these systems lies in their tunability—performance characteristics can be tailored by varying the type, concentration, and dispersion of nanoparticles within the matrix [2].

Among various nanocomposite systems, polydimethylsiloxane (PDMS) reinforced with silica (SiO_2_) nanoparticles has attracted considerable attention. PDMS is valued for its inherent flexibility, chemical stability, and ease of processing, while silica nanoparticles offer a means to significantly enhance its mechanical and functional performance [3,4].

Silica reinforced PDMS composites can be engineered with customizable properties by adjusting processing and crosslinking parameters during synthesis [5]. PDMS reinforced with silica also demonstrates excellent optical transparency and flexibility, along with strong resistance to bacterial colonization and chemical corrosion [6].

Incorporating silica into the PDMS matrix not only improves the coating’s mechanical strength but also promotes the formation of hierarchical surface structures. These micro/nanostructures contribute to enhanced durability and environmental resistance [7,8]. Notably, the well-dispersed silica particles facilitate the creation of air-trapping structures at the surface, thereby reducing the contact area with corrosive media. This barrier effect slows the diffusion of aggressive ions, markedly improving the coating’s corrosion resistance [9,10,11,12,13]. Additionally, the presence of rigid nanoparticles inhibits plastic deformation, thereby enhancing scratch and abrasion resistance—critical factors for extending the functional lifespan of protective coatings [14,15,16,17].

For applications, understanding the mechanical and tribological behavior of nanocomposite coatings at the nanoscale is essential. Techniques such as nanoindentation and nano-mechanical testing are highly effective for characterizing the local mechanical properties of thin films and coatings [18,19]. These methods enable precise quantification of hardness and complex modulus, offering valuable insights into both elastic and viscoelastic behavior [20,21,22].

According to a recent study, elevated temperatures enhance polymer chain mobility, reduce matrix stiffness, and modify nanoparticle-matrix interfacial bonding, which in turn influences the overall mechanical response of nanocomposites [23]. To probe time-dependent deformation mechanisms, nanoindentation tests incorporating a hold segment at peak load are often employed. This approach enables the assessment of viscoelastic and viscoplastic behavior under sustained loading conditions [1]. Such measurements are particularly important for polymer-based composites, where the interaction between the matrix and the nanoparticles plays a central role in defining mechanical performance. Temperature is a critical factor in this context, as elevated thermal conditions increase molecular mobility, reduce stiffness, and alter interfacial interactions within the composite structure [24,25]. Recent studies have shown that nanoindentation with hold periods is an effective method for evaluating the influence of strain rate, load, and temperature on the mechanical response of advanced materials [26,27,28,29,30].

In addition to mechanical testing, tribological characterization is essential for evaluating the coating’s wear resistance and frictional performance. The nanoscratch test, performed using a conical diamond tip under controlled loading, provides detailed information on resistance to localized deformation and the onset of coating failure [31]. This technique is useful for identifying critical load thresholds and analyzing scratch-induced deformation profiles. Complementary friction tests, such as linear reciprocating tribometry, measure the coefficient of friction under varying normal loads, offering further insight into the coating’s behavior under dynamic contact conditions [22].

This study examines the nanoscale mechanical and tribological behavior of a nanocomposite coating under testing conditions spanning temperatures from 24 °C to 120 °C. Nanoindentation was employed to measure key parameters such as nano-hardness and complex modulus, while nanoscratch and friction tests were used to assess the coating’s resistance to wear and its frictional characteristics under mechanical loading [31]. The integration of these experimental methods provides a comprehensive understanding of the coating’s performance, highlighting its potential for application in demanding environments under conditions where mechanical stability and surface functional properties are essential.

## 2. Materials and Methods

Coating deposition was performed on steel substrates. Anhydrous ethanol, acetone, and isopropyl alcohol (all procured from Sigma-Aldrich, St. Louis, MO, USA) were used. A silane-modified hydrophobic sil-ica nanoparticle powder (RX-50, Evonik, Piscataway, NJ, USA) along with a polydimethylsiloxane (PDMS) elastomer kit (Syl-gard 184, Dow Corning, Midland, MI, USA), served as the primary components for the nanocomposite coating formulation.

Prior to coating, the steel substrates underwent a standardized cleaning procedure involving degreasing followed by ultrasonic cleaning with acetone for 20 min at room temperature. The substrates were then sequentially rinsed with isopropyl alcohol, ethanol, and deionized (DI) water to eliminate residual surface contaminants. After cleaning, the substrates were dried in air at ambient temperature.

**Figure 1:**
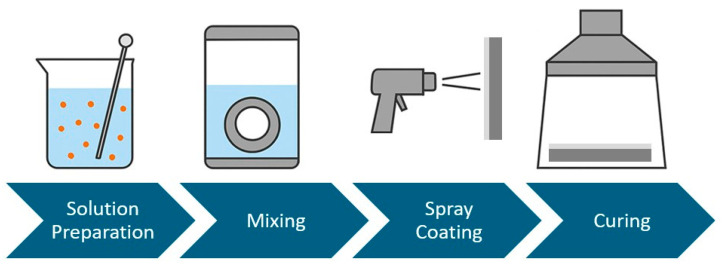
Schematic illustration of the fabrication process for the superhydrophobic nanocomposite coating.

The nanocomposite coating was prepared via a multi-step dispersion and mixing process. Initially, 1.7 g of PDMS was dissolved in 11 g of toluene using an ultrasonic device (UC-100, Cole-Parmer, Vernon Hills, IL, USA) for 1 min. Separately, 2 g of silica nanoparticles were dispersed in 10 g of toluene using a planetary centrifugal mixer (AR-100, Thinky, Laguna Hills, CA, USA) for 30 s. The two mixtures were then combined and homogenized for an additional 30 s. Subsequently, 0.17 g of curing agent was added, followed by a final 30 s of mixing and 30 s of defoaming to remove entrapped air. The resulting nanocomposite solution was applied to the substrates via spray coating and cured at ambient conditions in a fume hood for 72 h to ensure complete PDMS cross-linking. Figure 1 illustrates the key steps in the fabrication process of the nanocomposite coating. Toluene was chosen as the solvent due to its compatibility with both PDMS and silica, promoting uniform dispersion and minimizing nanoparticle agglomeration. During curing, the samples were exposed to ambient laboratory humidity with continuous airflow from the fume hood. The environment was maintained clean and free of dust to minimize contamination and ensure reproducibility. To achieve a uniform suspension, a planetary centrifugal mixer was employed to apply high shear forces capable of disrupting particle clusters.

The static water contact angle was measured using a drop shape analyzer (DSA25E, Krüss, Matthews, NC, USA) with 10 µL DI water droplets. Surface morphology was examined using scanning electron microscopy (SEM, JSM-7500F, JEOL, Peabody, MA, USA), and surface topography was further characterized by atomic force microscopy (AFM, Park NX10, Park Systems Co., Santa Clara, CA, USA). Mechanical properties were evaluated using nanoindentation with a TI 980 TriboIndenter (Bruker, Eden Prairie, MN, USA) equipped with a three-sided Berkovich diamond tip. All indentation tests were conducted under a maximum load of 1500 μN. A nanoscratch test was also con-ducted using the TI 980 TriboIndenter equipped with a standard conical diamond tip, featuring a nominal radius of 4–6 µm and an included angle of 60°. The scratch was performed at a constant speed of 1 µm/s over a total length of 10 µm under a normal load of 80 µN. A normal load of 80 µN was selected to ensure that the applied force remained within the elastic–plastic deformation regime of the nanocomposite coating, avoiding complete coating failure or substrate interference. Thickness measurements of the coating were conducted using a digital gauge (El-cometer, Manchester, UK).

## 3. Results

**Figure 2:**
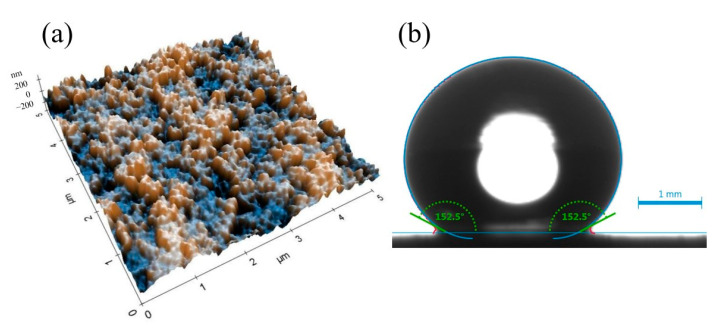
(**a**) an AFM image of the nanocomposite coating; (**b**) A static contact angle image for a water droplet of 10 µL on the superhydrophobic nanocomposite coating.

**Figure 3:**
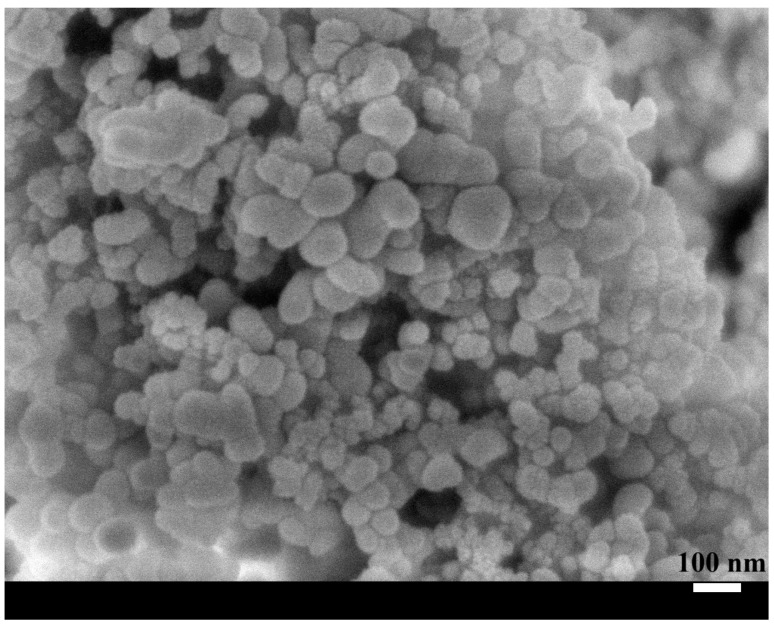
An SEM image of the nanocomposite coating.

**Figure 4:**
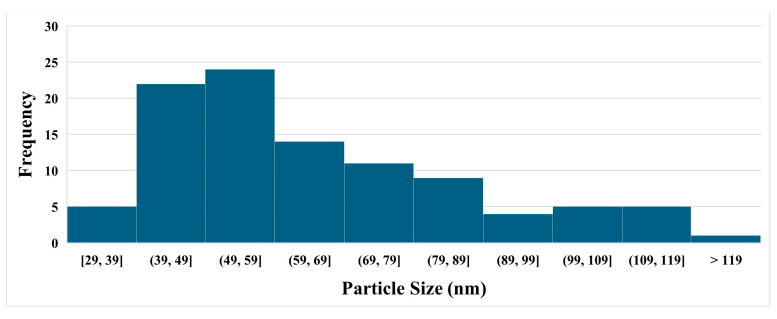
Size distribution histogram of silica nanoparticles of the nanocomposite coating.

**Figure 5:**
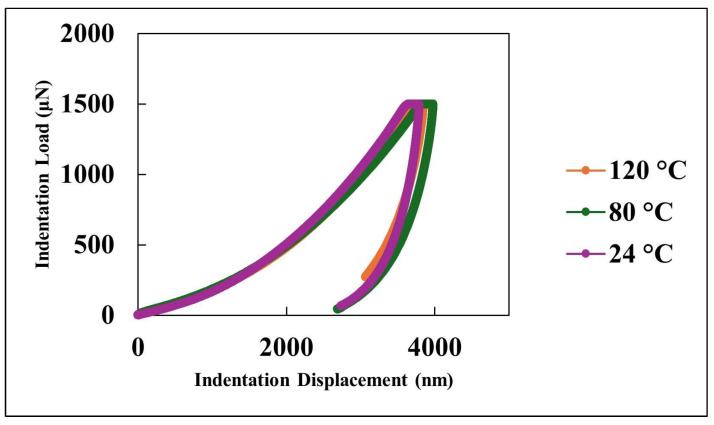
Indentation load–displacement curves at various temperatures.

Figure 2 illustrates the surface morphology and wettability characteristics of the nanocomposite coating. The atomic force microscopy (AFM) image (Figure 2a) reveals a hierarchical surface architecture, characterized by microscale roughness arising from nanoparticle agglomeration and finer nanoscale features attributed to individual silica particles. This dual-scale topography is instrumental in enhancing surface hydrophobicity by minimizing the solid–liquid contact area and promoting the Cassie–Baxter wetting regime. Correspondingly, the static water contact angle measurement (Figure 2b) demonstrates a high contact angle of 152.5°, confirming the coating’s excellent water-repellent properties. This pronounced hydrophobic behavior results from the synergistic effect of the chemical composition and multiscale surface roughness, which together facilitate the entrapment of air pockets beneath water droplets, thereby reducing adhesion and enabling superhydrophobic performance. The scanning electron microscopy (SEM) image shown in Figure 3 provides further insight into the surface structure of the nanocomposite coating. Figure 4 presents the size distribution histogram of silica nanoparticles within the coating. A well-distributed network of silica nanoparticles is evident, forming an interconnected framework that contributes to the coating’s textured morphology. This structure not only reinforces mechanical interlocking at the surface but also enhances functional properties such as hydrophobicity and abrasion resistance. The uniform distribution of silica within the PDMS matrix is critical for ensuring both performance consistency and structural integrity across the coating surface. Figure 5 presents the load–displacement curves obtained from nanoindentation tests conducted across a range of temperatures. In all cases, the penetration depth increases proportionally with the applied load, indicating a stable and predictable mechanical response under thermal variation. The presence of consistent hysteresis loops during the loading–unloading cycles highlights the coating’s ability to dissipate mechanical energy effectively. Importantly, the uniformity of these loops across the tested temperature range suggests that the coating maintains its viscoelastic energy dissipation characteristics despite thermal fluctuations. The consistent viscoelastic energy dissipation observed across the tested temperature range underscores the coating’s potential for applications requiring mechanical reliability under thermal variation, although other mechanical properties such as hardness and modulus do exhibit temperature sensitivity.

**Figure 6:**
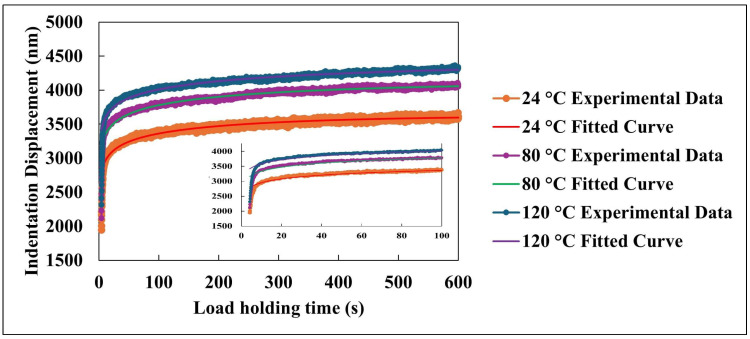
Experimental and fitted indentation displacement behavior at selected temperature. The inset highlights the initial ~100 s of load holding time to emphasize transient deformation behavior.

Figure 6 presents the time-dependent indentation displacement of the nanocomposite coating subjected to a constant load of 1500 µN at three different temperatures: 24 °C, 80 °C, and 120 °C. The experimental data capture the material’s deformation behavior over a 600 s hold segment during instrumented nanoindentation testing. These displacement profiles reflect the combined viscoelastic and viscoplastic responses of the material under sustained mechanical loading, particularly at elevated temperatures. At the beginning of the hold period, a rapid increase in indentation depth is observed, indicating the material’s immediate deformation response to the applied load. This initial phase is governed by elastic deformation and early viscoplastic flow, resulting in a steep rise in displacement. As time progresses, the rate of indentation gradually decreases, entering a more stable regime characterized by a slower, nearly linear increase in displacement. This trend suggests a dynamic equilibrium between the time-dependent deformation mechanisms and the material’s intrinsic resistance to further penetration under constant loading.

To quantitatively describe the indentation behavior, a phenomenological model was applied to fit the experimental data. The fitted curves, represented in Figure 6, are described by the following equation [32,33]:

indentation displacement = a + bt + cln(dt + 1)(1)

where t is the hold time in seconds, and a, b, c, and d are fitting coefficients. This model demonstrated excellent agreement with experimental results, yielding high correlation coefficients across all temperatures and effectively capturing the complex time dependent deformation behavior.

Each parameter in the model corresponds to a specific aspect of the indentation response. The coefficient a represents the initial indentation depth at the onset of the hold period, reflecting the instantaneous elastic and early viscoplastic deformation. Coefficient b captures the linear time-dependent component of the displacement, associated with the steady state progression of deformation under constant load. The coefficient c describes the magnitude of the logarithmic term, which effectively models the rapid, nonlinear displacement during the early stages of the hold period. Lastly, coefficient d controls the rate at which the indentation response transitions from the initial rapid phase to the subsequent slower regime.

Temperature plays a significant role in influencing the indentation behavior. As shown in Figure 6, there is a clear positive correlation between temperature and total indentation displacement. At 120 °C, the maximum displacement reaches approximately 4300 nm at 600 s, while at 24 °C, the displacement is reduced to about 3600 nm. Additionally, the slope of the displacement–time curves, representing the rate of indentation, shows a strong dependence on temperature. Higher temperatures result in more pronounced displacement rates during the initial ~100 s, indicating an acceleration of deformation mechanisms due to increased molecular mobility and reduced matrix stiffness.

All indentation-time profiles exhibit a distinct two stage deformation pattern: (1) an initial rapid rise in displacement within the first ~100 s, corresponding to the unstable primary indentation phase; and (2) a slower, more linear increase characterizing the secondary phase of stable deformation. The phenomenological model captures both regimes effectively. The logarithmic component models the early stage, nonlinear response, which is crucial for understanding the initial mechanical behavior and predicting long term performance of coatings in practical applications. The linear term, on the other hand, reflects the steady-state deformation rate, critical for evaluating the coating’s long-term resistance to penetration and degradation. The proposed model provides a predictive framework for characterizing the time-dependent mechanical response of polymer-based nanocomposite coatings under constant loading and elevated temperatures. By isolating and quantifying the contributions of rapid initial deformation and slower steady-state progression, the model offers valuable insight into the underlying viscoelastic and viscoplastic mechanisms governing the performance of thin films and surface coatings. These insights can inform material design strategies aimed at optimizing mechanical stability and service life under thermomechanical stress conditions.

**Figure 7:**
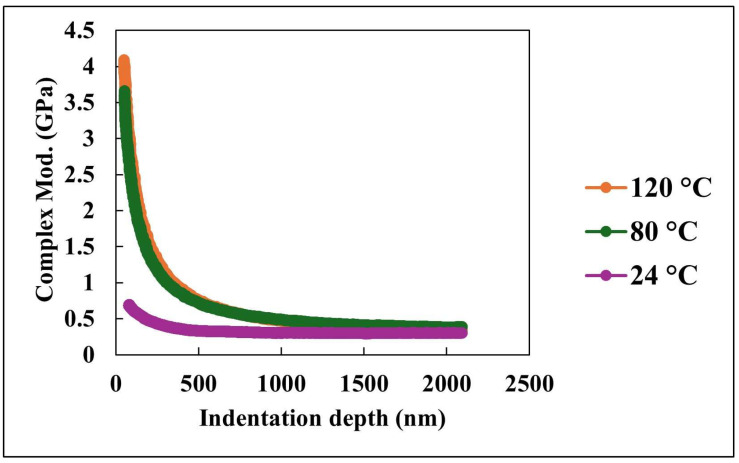
Complex modulus versus indentation depth at various temperatures.

**Figure 8:**
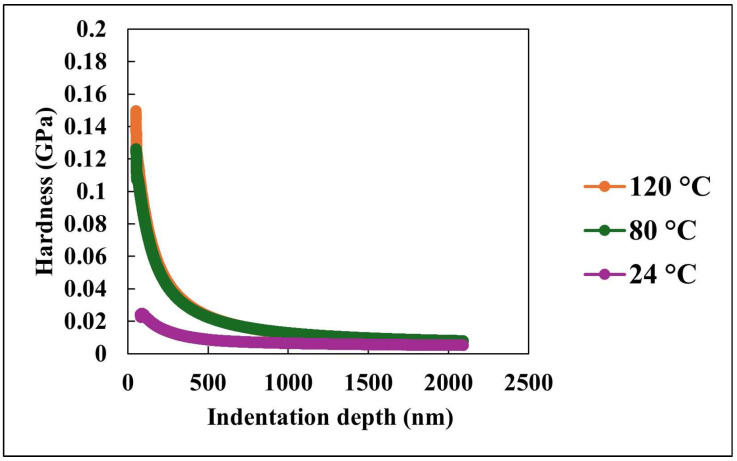
Hardness versus indentation depth at various temperatures.

Figure 7 and Figure 8 collectively elucidate the viscoelastic and mechanical responses of the nanocomposite coating as functions of indentation depth and temperature, as characterized by nano-mechanical analysis. Figure 7 displays the variation in complex modulus with indentation depth at three temperatures: 24 °C, 80 °C, and 120 °C. The complex modulus is defined as the combination of the storage modulus, which represents the elastic (energy-storing) response, and the loss modulus, which represents the viscous (energy-dissipating) response of the material. It serves as a comprehensive indicator of the material’s resistance to deformation under dynamic loading conditions. At shallow indentation depths, the coating exhibits its highest complex modulus, particularly at 24 °C, suggesting a stiffer and more structurally reinforced surface layer. As the indentation depth increases, the complex modulus decreases across all tested temperatures, indicating a transition from surface-dominated mechanical response to bulk-dominated behavior. This depth-dependent reduction in modulus is especially pronounced at elevated temperatures, where thermal softening accelerates the decline, highlighting the coating’s sensitivity to temperature.

Notably, the complex modulus curves for all temperatures converge at depths beyond approximately 2000 nm. This convergence suggests that, beyond the influence of the surface architecture, the bulk material exhibits a more uniform and compliant viscoelastic response. The observed decline in complex modulus with both increasing depth and temperature can be attributed to the thermally induced softening of the polymer matrix and the diminishing effectiveness of surface-level reinforcement mechanisms. These results underscore the critical role of engineered surface structures in enhancing stiffness at ambient conditions, while also revealing the limitations of these enhancements under elevated thermal stress.

Figure 8 complements the modulus analysis by illustrating the variation in hardness with indentation depth at the same set of temperatures. Hardness, which quantifies the material’s resistance to localized plastic deformation, exhibits a similar decreasing trend with depth. At 24 °C, the coating exhibits a relatively lower initial hardness compared to higher temperatures. As temperature increases to 80 °C and 120 °C, both the initial hardness values and their resistance to depth-dependent degradation decline significantly. This behavior is indicative of thermally activated softening of the polymer matrix, which facilitates plastic flow under the indenter.

At greater depths, the hardness values for all temperatures converge to a narrow range below 0.03 GPa. This trend parallels the complex modulus data, reinforcing the conclusion that the bulk mechanical response is largely homogeneous and less influenced by surface features or thermal effects. The elevated hardness at shallow depths, especially at lower temperatures, highlights the significant contribution of surface nano/microstructural architecture in resisting deformation. These features likely enhance mechanical interlocking and restrict polymer chain mobility at the surface, thereby improving local resistance to plastic indentation.

Figure 7 and Figure 8 provide a comprehensive view of the nanocomposite coating’s mechanical behavior under varying temperature and mechanical loading conditions. The depth-dependent decline in both complex modulus and hardness emphasizes the importance of surface engineering in enhancing performance at the nanoscale. Meanwhile, the temperature-dependent trends reveal the inherent limitations of these enhancements under elevated temperature environments. At ambient temperature, the PDMS matrix retains a high degree of flexibility, which may lead to reduced resistance to localized indentation. In contrast, elevated temperatures may promote partial softening of the matrix while simultaneously increasing the exposure of embedded silica nanoparticles at the surface. This enhanced nanoparticle contribution may locally reinforce the surface, resulting in higher initial hardness values.

**Figure 9:**
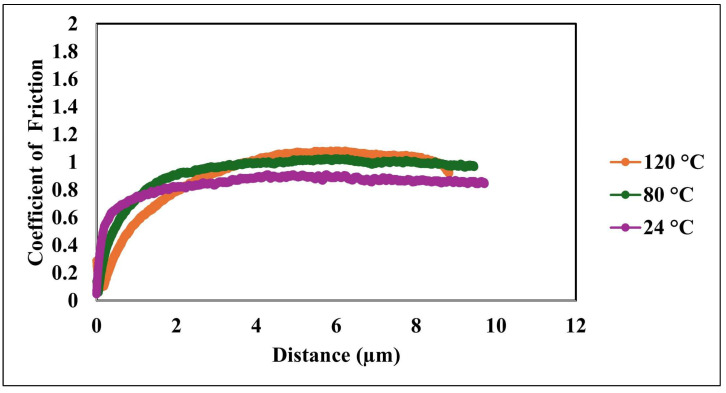
Coefficient of friction (COF) as a function of sliding distance for the nanocomposite coating at various temperatures.

**Figure 10:**
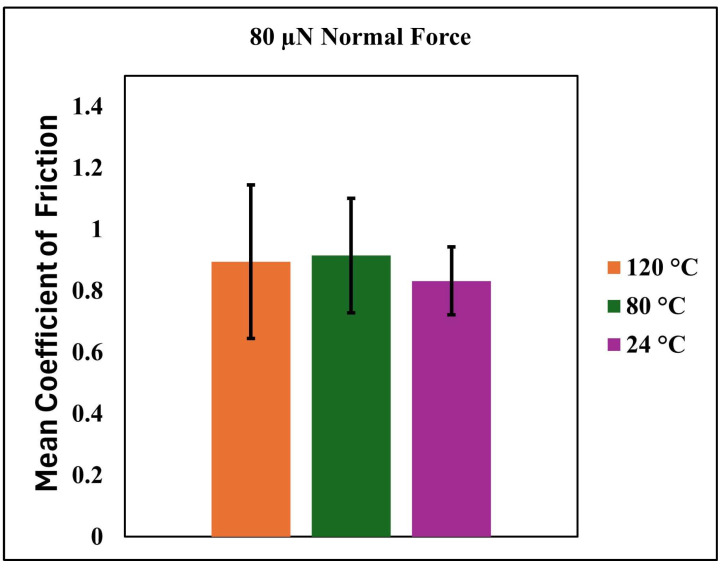
Mean coefficient of friction of the nanocomposite coating under an 80 µN normal force at various temperatures.

Figure 9 and Figure 10 provide a detailed assessment of the nanocomposite coating’s tribological performance under varying thermal conditions, highlighting the influence of temperature on frictional behavior and wear characteristics. Figure 9 illustrates the evolution of the coefficient of friction (COF) with sliding distance for the nanocomposite coating tested at three temperatures: 24 °C, 80 °C, and 120 °C. This analysis offers critical insight into the coating’s frictional behavior under thermal and mechanical loads, which is vital for evaluating its suitability in high-temperature applications involving dynamic contact. Across all temperatures, the COF displays a typical trend characterized by an initial rise followed by a plateau, corresponding to the running-in phase and subsequent steady-state sliding. During the running-in phase, surface asperities interact and conform to each other, leading to increased real contact area and frictional stabilization. At 24 °C, the COF begins at a relatively low value and stabilizes around 0.6 after approximately 4 µm of sliding, suggesting moderate frictional resistance under ambient conditions. This behavior can be attributed to the compliant PDMS matrix, which reduces interfacial shear stress through surface flexibility. At 80 °C, a similar trend is observed; however, the steady-state COF reaches a slightly higher value, reflecting increased interfacial resistance. This increase is likely due to the thermal softening of the PDMS matrix, which may expose more silica nanoparticles and increase surface roughness, thereby enhancing friction. At 120 °C, the COF exhibits a more pronounced initial increase and stabilizes near 1.0, indicating a substantial rise in frictional resistance. This elevated COF suggests that significant changes occur in the surface and subsurface structure at high temperatures, potentially including matrix degradation, increased prominence of the rigid silica phase, and enhanced mechanical interlocking at the contact interface. Additionally, thermal expansion and changes in viscoelastic properties may increase energy dissipation during sliding, further contributing to higher friction.

The comparative analysis across temperatures demonstrates a clear temperature-dependent trend in COF magnitude, underscoring the necessity of accounting for thermal effects when designing coatings for elevated-temperature applications. Importantly, while the magnitude of the COF increases, the overall frictional profile remains stable, suggesting that the coating retains mechanical robustness and a consistent sliding response even under thermal stress.

Figure 10 complements this analysis by presenting the mean COF values measured under a constant normal force of 80 µN at the same three temperatures. The data confirm the general trend observed in Figure 9, with COF increasing as temperature rises. Interestingly, the highest mean COF is recorded at 80 °C, suggesting that this temperature may represent a transitional point where the matrix softening and the exposure of the silica reinforcement phase are both at a maximum. At 120 °C, although the COF remains elevated, a slight decrease is observed compared to 80 °C. This may be due to further softening or partial viscous flow of the matrix, which could reduce the effective contact area or change the dominant friction mechanism from abrasive to more adhesive or viscoelastic in nature. Figure 9 and Figure 10 offer a comprehensive view of the nanocomposite coating’s tribological response across a realistic operating temperature range. The observed trends reflect a complex interplay between surface roughness evolution, polymer softening, and interfacial mechanics. These findings are instrumental for optimizing the coating formulation and predicting long-term performance under combined thermal and mechanical loads.

## 4. Conclusions

This study presents a comprehensive investigation into the mechanical and tribological behavior of a PDMS–silica nanocomposite coating over a temperature range of 24 °C to 120 °C. Nanoindentation analyses revealed a pronounced dependence of mechanical properties on both indentation depth and temperature. Specifically, the complex modulus and hardness exhibited a decreasing trend with increasing depth, reflecting the transition from surface-dominated to bulk material response and the diminishing influence of surface microstructure. Elevated temperatures further accentuated this effect, significantly reducing the coating’s stiffness and resistance to deformation, thereby highlighting its thermal sensitivity. Time-dependent indentation measurements revealed distinct two-stage deformation behavior, characterized by an initial rapid displacement followed by a slower, steady-state phase. These experimental trends were accurately captured using a phenomenological time-dependent model, which demonstrated strong predictive capability across all test conditions. The model not only quantified key aspects of the viscoelastic and viscoplastic responses but also provided critical insight into the underlying deformation mechanisms, offering a valuable tool for forecasting mechanical performance under thermomechanical loading. Tribological testing reinforced the temperature-dependent nature of the coating’s behavior. The coefficient of friction increased from approximately 0.83 at 24 °C to nearly 0.9 at 120 °C, suggesting enhanced interfacial shear stress and changes in surface interaction mechanisms. Based on the experimental results, the coating demonstrated stable mechanical and tribological performance up to 120 °C. This temperature is therefore considered to be near the upper bound of the coating’s effective operating range for practical applications.

Overall, the integration of mechanical, viscoelastic, and surface metrology and characterization techniques has yielded a holistic understanding of the nanocomposite coating’s performance under varying thermal and mechanical conditions. The findings underscore the critical role of surface architecture in enhancing durability and functional performance, particularly at various temperatures. The validated time-dependent model, along with the comprehensive experimental data, provides a solid foundation for the future design, optimization, and deployment of PDMS–silica nanocomposite coatings in high-performance, thermally demanding applications.

## Data Availability

The original contributions presented in this study are included in the article. Further inquiries can be directed to the corresponding author.

## References

[1] Buehler M.J., Misra A. (2019). Mechanical behavior of nanocomposites. MRS Bull..

[2] Sebastian D., Yao C.-W. (2020). Effect of poly(dimethylsiloxane) binder in a silica-based superhydrophobic coating on mechanical properties, surface roughness, and wettability. MRS Commun..

[3] Sebastian D., Yao C.W., Lian I. (2020). Multiscale corrosion analysis of superhydro phobic coating on 2024 aluminum alloy in a 3.5 wt.% NaCl solution. MRS Commun..

[4] Wang Z., Yang A., Tan X., Tu Y., Sabin S., Xiang P., Chen X. (2020). A veil-over-sprout micro-nano PMMA/SiO_2_ superhydrophobic coating with impressive abra sion, icing, and corrosion resistance. Colloids Surf. A.

[5] Cordoba A., Cauich-Rodríguez J.V., Vargas-Coronado R.F., Velázquez-Castillo R., Esquivel K. (2024). A Novel In Situ Sol-Gel Synthesis Method for PDMS Composites Reinforced with Silica Nanoparticles. Polymers.

[6] Guo X., Di Y., Liang Q., Li P., Lv J., Tian Y., Li Q., Jiang L., Xu C., Zhang Z. (2023). Inorganic–Organic Silica/PDMS Nanocomposite Antiadhesive Coating with Ultrahigh Hardness and Thermal Stability. ACS Appl. Mater. Interfaces.

[7] Wang Y., Li M., Lv T., Wang Q., Chen Q., Ding J. (2015). Influence of different chemical modifications on the icephobic properties of superhydrophobic surfaces in a condensate environment. J. Mater. Chem. A.

[8] Zaman Khan M., Militky J., Petru M., Tomková B., Ali A., Tören E., Perveen S. (2022). Recent advances in superhydrophobic surfaces for practical applications: A review. Eur. Polym. J..

[9] Li C., Ma R., Du A., Fan Y., Zhao X., Cao X. (2019). One-step fabrication of bionic superhydrophobic coating on galvanized steel with excellent corrosion resistance. J. Alloy. Compd..

[10] Zhang S.J., Cao D.L., Xu L.K., Lin Z.F., Meng R.Q. (2020). Fabrication of a superhydrophobic polypropylene coating on magnesium alloy with improved corrosion resistance. Int. J. Electrochem. Sci..

[11] Gong A., Zheng Y., Yang Z., Guo X., Gao Y., Li X. (2021). Spray fabrication of superhydrophobic coating on aluminum alloy for corrosion mitigation. Mater. Today Commun..

[12] Huang J., Lou C., Xu D., Lu X., Xin Z., Zhou C. (2019). Cardanol-based polybenzo xazine superhydrophobic coating with improved corrosion resistance on mild steel. Prog. Org. Coat..

[13] Deyab M.A. (2020). Anticorrosion properties of nanocomposites coatings: A critical review. J. Mol. Liq..

[14] Yu J., Qin L., Hao Y., Kuang S., Bai X., Chong Y.-M., Zhang W., Wang E. (2010). Vertically Aligned Boron Nitride Nanosheets: Chemical Vapor Synthesis, Ultraviolet Light Emission, and Superhydrophobicity. ACS Nano.

[15] Li Y., Zhang L., Li C. (2020). Highly transparent and scratch resistant polysiloxane coatings containing silica nanoparticles. J. Colloid Interface Sci..

[16] Dong D., Chen X.H., Xiao W.T., Yang G.B., Zhang P.Y. (2009). Preparation, and properties of electroless Ni–P–SiO_2_ composite coatings. Appl. Surf. Sci..

[17] Gutsev D., Antonov M., Hussainova I., Grigoriev A.Y. (2013). Effect of SiO_2_ and PTFE additives on dry sliding of NiP electroless coating. Tribol. Int..

[18] Arora G., Pathak H. (2021). Nanoindentation characterization of polymer nanocomposites for elastic and viscoelastic properties: Experimental and mathematical approach. Compos. Part C Open Access.

[19] Karimzadeh A., Koloor S.S.R., Ayatollahi M.R., Bushroa A.R., Yahya M.Y. (2019). Assessment of Nano-Indentation Method in Mechanical Characterization of Heterogeneous Nanocomposite Materials Using Experimental and Computational Approaches. Sci. Rep..

[20] Liang X. (2023). Visualization of Nanomechanical Properties of Polymer Composites Using Atomic Force Microscopy. Polym. J..

[21] Bor B., Giuntini D., Domènech B., Swain M.V., Schneider G.A. (2019). Nanoindentation-based study of the mechanical behavior of bulk supercrystalline ceramic-organic nanocomposites. J. Eur. Ceram. Soc..

[22] Ryou H., Romberg E., Pashley D.H., Tay F.R., Arola D. (2012). Nanoscopic dynamic mechanical properties of intertubular and peritubular dentin. J. Mech. Behav. Biomed. Mater..

[23] Klonos P.A., Christodoulou E., Katsika T.C., Papoulia C., Chrissafis K., Kyritsis A., Bikiaris D.N. (2022). Thermal transitions, interfacial interactions, and molecular mobility in nanocomposites based on poly(l,d-lactic acid) and fumed silica nanoparticles. J. Therm. Anal. Calorim..

[24] Shan L., Tan C.Y., Shen X., Ramesh S., Zarei M.S., Kolahchi R., Hajmohammad M.H. (2023). The effects of nano-additives on the mechanical, impact, vibration, and buckling/post-buckling properties of composites: A review. J. Mater. Res. Technol..

[25] Mahmoud Z.H., Al-Salman H.N.K., Kianfar E. (2024). Nanoindentation: Introduction and applications of a non-destructive analysis. Nano TransMed.

[26] Wang J., Yang C., Liu Y., Li Y., Xiong Y. (2022). Using Nanoindentation to Characterize the Mechanical and Creep Properties of Shale: Load and Loading Strain Rate Effects. ACS Omega.

[27] Yang C., Liu Y., Wang J., Wu D., Liu L., Su Z., Xiong Y. (2023). Application of nanoindentation technique in mechanical charac-terization of organic matter in shale: Attentive issues, test protocol, and technological prospect. Gas Sci. Eng..

[28] Zhang P., Zhang D., Zhao J. (2024). Control of fracture toughness of kerogen on artificially-matured shale samples: An energy-based nanoindentation analysis. Gas Sci. Eng..

[29] Huang H., Zhang W., Shi H., Ni J., Ding L., Yang B., Zheng Y., Li X. (2024). Experimental investigation of microscale mechanical alterations in shale induced by fracturing fluid contact. Gas Sci. Eng..

[30] Wang J., Dziadkowiec J., Liu Y., Jiang W., Zheng Y., Xiong Y., Peng P.A., Renard F. (2024). Combining atomic force microscopy and nanoindentation helps characterizing in-situ mechanical properties of organic matter in shale. Int. J. Coal Geol..

[31] Skarmoutsou A., Charitidis C.A., Gnanappa A.K., Tserepi A., Gogolides E. (2012). Nanomechanical and nanotribological properties of plasma nanotextured superhydrophilic and superhydrophobic polymeric surfaces. Nanotech. Nol..

[32] Vandamme M., Ulm F.-J. (2009). Nanogranular origin of concrete creep. Proc. Natl. Acad. Sci. USA.

[33] Liu K., Jin Z., Zeng L., Ostadhassan M., Xu X. (2021). Understanding the creep behavior of shale via nano-DMA method. Energy Rep..

